# Regulations, Open Data and Healthcare Innovation: A Case of MSK-IMPACT and Its Implications for Better Cancer Care

**DOI:** 10.3390/cancers13143448

**Published:** 2021-07-09

**Authors:** Takaharu Jibiki, Hayato Nishimura, Shintaro Sengoku, Kota Kodama

**Affiliations:** 1 Department of Innovation Science, School of Environment and Society, Tokyo Institute of Technology, Tokyo 108-0023, Japan; jibiki.t.aa@m.titech.ac.jp (T.J.); nishih@riken.jp (H.N.); 2 Policy Planning Division, RIKEN, Saitama 351-0198, Japan; 3 Life Style by Design Research Unit, Institute for Future Initiatives, University of Tokyo, Tokyo 113-0033, Japan; 4 Graduate School of Technology Management, Ritsumeikan University, Osaka 567-8570, Japan; kkodama@fc.ritsumei.ac.jp

**Keywords:** new drug development, next-generation sequencing (NGS), open data, regulatory reform, tumor profiling test

## Abstract

**Simple Summary:**

The advancement in both science and technology has contributed to the development of novel diagnostic technologies; such technologies enable medical practitioners to diagnose diseases that could not be previously detected. However, in order to translate new technologies into practical applications, various types of challenges need to be overcome. To address these challenges, including those in clinical management and regulatory science, healthcare policies have been constantly implemented to promote the practical application of outcomes generated by healthcare innovation. This study conducted comparative analyses of three tumor profiling tests approved by the U.S. Food and Drug Administration (FDA) in 2017, hypothesizing that the FDA’s regulatory reforms, early application of new technologies to both research and clinical settings, and open data accumulated as a result of large-scale research programs have promoted new drug development in oncology. The study then discussed the implications potentially suggested by the outcomes and challenges of the three tests.

**Abstract:**

This study investigated a case of Memorial Sloan Kettering-Integrated Mutation Profiling of Actionable Cancer Targets (MSK-IMPACT), a tumor profiling test approved by the U.S. Food and Drug Administration (FDA) in 2017, to examine what factors would contribute to healthcare innovation. First, we set the following three parameters to observe cases: (i) the FDA regulatory reforms, (ii) early application of new technologies, such as next-generation sequencing (NGS), to both research and clinical settings, and (iii) accumulation of open data. Then, we performed a comparative analysis of MSK-IMPACT with FoundationOne CDx and Oncomine Dx Target Test, both of which were FDA-approved tumor profiling tests launched in 2017. As a result, we found that MSK-IMPACT secures neutrality as a non-profit organization, achieves the active incorporation of basic research results, and performs superiorly in clinical operations, such as patient enrollment. On the contrary, we confirmed that FoundationOne CDx was the most prominent case in terms of the number of new drugs and expanded indications approved in which the FDA’s expedited approval programs were considerably utilized. Consequently, to uncover the full potential of MSK-IMPACT, it is suggested that more intersectoral collaborative activities between various healthcare stakeholders, in particular, pharmaceutical companies, for driving clinical development must be carried out based on an organizational framework that facilitates collaboration.

## 1. Introduction

### 1.1. Next-Generation Sequencing (NGS) for Advanced Medicine

As genome science advances, personalized medicine, which would enable tailor-made medical solutions based on personal biological information, is expected to become a reality. The 2015 State of the Union Address announced that the United States would make nationwide efforts to realize the Precision Medicine Initiative, which sought to establish healthcare, considering differences among individuals that could be caused by genes, environment, lifestyle, and so forth [1]. The initiative covered a variety of issues, such as the development and delivery of cancer care, establishment of a nationwide research cohort leveraging over 1 million volunteers, development of new validation methods for Next Generation Sequencing (NGS) instruments and data sharing platforms, and regulatory reforms [1].

NGS is known to have dramatically reduced sequencing costs [2] and has contributed to the practice of large collaborative research projects worldwide. Since this technology has enabled researchers to efficiently analyze the genetic information of target samples at a reasonable cost, the application of NGS now ranges from analysis of genetic mutations of cancer patients to that of information on microbial samples, such as the human microbiome. NGS can surely extend the frontier of healthcare by practically helping researchers realize the application of personalized medicine in a clinical setting.

Although new technologies, such as NGS, allow scientists to explore new research areas, they do not necessarily ensure safety due to the lack of data and precedents. Therefore, the development and further application of new therapeutic options to a clinical setting based on bioinformatics requires, to a certain extent, regulatory efforts by relevant authorities that can simultaneously ensure both the safety and efficacy. In addition, it is recommended that biological data necessary for the development of new therapeutic options be open to the public. It is reasonable to assume that the researchers can be encouraged to access a database of biological information if they can use it at any given time. It is also recommended that such data be regularly updated, with a certain degree of standardization and compatibility between different datasets. In general, no researcher wants to use either obsolete or unstandardized data without their compatibility with other datasets, as these are factors that can affect the quality of the scientific research.

### 1.2. Regulatory Reforms for the Pharmaceutical Industry

Healthcare innovation can be induced by implementing efficient regulations [3]. This can apply not only to pharmaceuticals, but also to new technological fields, such as mobile health (mHealth). Onodera et al. 2018 revealed that the regulatory reforms implemented by the FDA indirectly contributed to the increase in the number of FDA-cleared mobile medical apps during the mid-2010s [4]. This implies that regulations can even stimulate innovation in such an emerging field with uncertainly if they are appropriately implemented to support innovators. The question here is to what extent pharmaceutical regulations in the United States have facilitated innovation in terms of conventional pharmaceutical development and commercialization.

The U.S. Food and Drug Administration (FDA) has started making regulatory reforms in drug approvals with a certain degree of organizational efforts since the early 1980s. Such reforms are supposed to have partly contributed to promoting innovation. For example, the distribution of orphan drugs among all FDA-approved drugs increased from 17 percent (1984–1988) to 31 percent (2004–2008) after the Orphan Drug Act of 1983, which was enacted at the earliest stage of the regulatory reforms by the FDA [5]. Moreover, the proportion of approved drugs that qualified for the FDA’s expedited approval programs (i.e., Orphan Drug Act (1983) [6], Fast Track Designation (1988) [7], Accelerated Approval Program (1992) [8], and Breakthrough Therapy Designation (2012) [9]) has increased from 1984 to 2018 [10]. For example, 22 out of the 39 FDA-approved drugs in 2012 were reported to have utilized such programs [11].

On the other hand, Golodner et al. 1998 raised a concern that expedited approval programs, which were intended to shortcut a drug review process toward approval, would deliver “dangerous or unnecessary drugs” to the users [12]. Since data obtained through the use of such programs rely on early-stage clinical trials, the quantity of clinical evidence tends to be limited and unstable [11]. The trade-off between the speed of the approval process and the efficacy of a drug candidate has remained a critical issue for the FDA to overcome.

Meanwhile, the FDA has succeeded in shortening the drug review time from more than 3 years in 1983 to less than 1 year in 2017 [10]; the major strategies were (1) to collect user fees from pharmaceutical companies to raise funds needed to review the increasing number of new drug applications under the Prescription Drug User Fee Act (PDUFA) of 1992 [10,13], and (2) to encourage the use of surrogate measures for clinical trials [10,14]. Nonetheless, the total time needed for clinical trials, which ranges from the application for Investigational New Drug (IND) to the FDA approval, has not been reduced from 1986 to 2017 [10]; it averaged at approximately 8 years during this period [10]. As expedited programs were utilized for the development of drugs for rare diseases, recruitment challenges for clinical trials and therapeutic challenges, both of which were found to be the typical difficulties specific to the drug development for such diseases, have arisen; these may have prolonged the overall clinical development time [10].

In addition, a series of regulatory reforms have not necessarily led to a dramatic increase in the number of new drugs approved between 1982 and 2018 [10]. The mean number of new drug approvals per annum, including those for biologics, between 1990 and 1999, was 34 [10]. However, the number remained at 41 between 2010 and 2018 [10]. To summarize, the FDA has taken certain actions to implement the regulatory reforms for the past three decades while undergoing some occasional setbacks.

### 1.3. Open Data and Healthcare Innovation

Previous research on the association between healthcare innovation and open data has been scarce. Goodsell et al. 2019 clarified in their study that the Protein Data Bank (PDB) archive, which was the “first open-access digital data resource” that provided researchers with data on three-dimensional (3D) protein structures, has contributed to new drug development since its establishment in 1971 [15]. The PDB database, which allowed open access to approximately 6000 protein structures, contributed to the FDA’s new drug approval of “88% of 210 new molecular entities” from 2010 to 2016 [15]. The PDB archive has grown dramatically over time through the accumulation of data on protein structures and other relevant topics. PDB users and data depositors, including a global expert community in structural biology, deposit data regarding protein structures into the archive [15]. Moreover, PDB data are updated on a weekly basis by integrating them with multiple external databases [15].

NGS technologies, in turn, generate biological data on target samples that a researcher intends to analyze. However, the contribution of such technologies to innovation is yet to be adequately discussed. Kahn et al. 2014 reported that the discussions were held on how NGS should be utilized for scientific research at the NGS for Cancer Drug Development conference held in Boston, USA, in September 2013 [16]. Participants from both the industry and academia discussed how they utilized data generated by NGS, such as the utilization of biomarker data for cancer drug development [16]. The use of “publicly available NGS data for target discovery,” along with the importance of “data integration” and “quality control,” were also discussed at this conference [16]. However, whether such data have contributed to facilitating innovation is yet to be thoroughly discussed.

### 1.4. Purpose of the Study

This study aimed to identify the institutional and organizational factors that can facilitate (or hinder) the development and dissemination of novel bioinformatics-based therapies. Considering the uniqueness of the product and its early practical utilization as a catalyst for an entry into cancer care services with NGS technologies, this study specifically focused on the case of Memorial Sloan Kettering-Integrated Mutation Profiling of Actionable Cancer Targets (MSK-IMPACT) to discuss how clinical sequencing and genomic cancer medicine could be promoted.

MSK-IMPACT, one of the first three FDA-approved tumor profiling tests launched in the market [17], is unique in that it was developed by the Memorial Sloan Kettering Cancer Center (MSKCC), a private cancer center located in Manhattan, New York City, USA; this was unlike FoundationOne CDx (Foundation Medicine, Inc., Cambridge, MA, USA) and Oncomine Dx Target Test (Life Technologies Corporation, Carlsbad, CA, USA), both of which were developed by companies and approved in the same year. MSK-IMPACT was a product developed by a hospital and had indeed been applied to a clinical setting before it was approved by the FDA as an in vitro diagnostic (IVD) test.

This study was centered around the following two questions: (i) How have the FDA’s regulatory reforms facilitated the development of new drug candidates identified by MSK-IMPACT? and (ii) how has MSK-IMPACT helped identify new drug candidates in oncology, leveraging open data accumulated through global research projects. To better answer these questions, this study particularly investigated the following regulatory and technological aspects: (i) FDA’s regulatory reforms and their outcomes, (ii) the contribution of publicly accessible open databases, specifically those based on the genetic mutations provided by cancer patients and established through large-scale research projects, and (iii) early application of new technologies (i.e., MSK-IMPACT) to both research and clinical settings. To ensure both fairness and objectiveness and to better clarify the outcomes of each panel test, we carried out a comparison between MSK-IMPACT and the other two panel tests, all of which were the first marketed products [17].

Based on the analysis of such comparisons, we then attempted to understand the characteristics and challenges associated with MSK-IMPACT by comparing them with those associated with FoundationOne CDx. Furthermore, we have also discussed how clinical sequencing in oncology should be further promoted to deliver and maximize the benefits of the technology in an efficient manner.

## 2. Materials and Methods

### 2.1. The Case

Following a review of the existing literature, this study sought to consider whether the FDA’s regulatory reforms have led to an early application of new technologies in both research and clinical settings, with a specific focus on the case of MSK-IMPACT. It also aimed to examine whether bioinformatics-driven innovation had been promoted in clinical sequencing in oncology as a result of the accumulation and utilization of publicly accessible open data on genetic information. Overall, research was conducted by referring to the public information released by the relevant organizations and employing a semi-structured interview with a key individual in the clinical oncology sequencing community. To offer a better understanding of the results of the research, Table 1 summarizes various types of relevant stakeholders and catalysts for cancer care innovation identified by the investigation of this study.

### 2.2. Document-Based Analysis

Considering the nature of the study, we mostly referred to qualitative information released by the FDA and MSKCC as well as to other relevant articles as the major sources of information.

For the first step of a literature search, this study employed the Patient, Intervention, Comparison, Outcome (PICO) framework for a preliminary search to gain a better understanding about the case, and to develop literature search strategies. Some of the typical search terms used were as follows: “cancer patients,” “MSK-IMPACT,” “FoundationOne CDx,” “Oncomine Dx Target Test,” and “new drug development.” Second, we hypothesized that (i) regulations, (ii) publicly accessible open data, and (iii) early application of new technologies induced by the regulations as key drivers of bioinformatics-driven innovation. After that, we performed database searches to obtain relevant articles that cover issues of the above 3 hypotheses; we performed each database search on Web of Science (https://www.webofscience.com/wos/woscc/basic-search, accessed on 7 March 2020) by using up to any of the 3 search terms at a time from the following: “bioinformatics,” “innovation,” “facilitate,” “facilitation,” “new drug development,” “NGS,” and “regulation.” As a result, we found 148 articles in total. Of these, we selected and examined 13 articles that were considered most relevant to the topics and hypotheses for this study. Furthermore, we conducted an issue-specific literature search on Scopus (https://www.scopus.com/search/form.uri?display=basic#basic, accessed on 21 June 2020) and Google Scholar (https://scholar.google.com/, accessed on 21 June 2020), focusing on a single issue relating to any of the above 3 hypotheses (i.e., a combination of issue-specific search terms “FDA Modernization Act” and “drug development”).

We also conducted a database search using PubMed (https://pubmed.ncbi.nlm.nih.gov/, accessed on 3 January 2019) and ClinicalTrials.gov (https://clinicaltrials.gov/, accessed on 3 January 2019) to gain quantitative implications and sought to confirm the number of scientific publications relating to data on genetic information and the number of clinical trials relating to cancer genomic medicine from the early 2000s to the late 2010s. In order to confirm the former, we used the search terms “GWAS” (genome-wide association study) and “SNP” (single nucleotide polymorphism) to separately investigate the numbers of publications regarding these technological issues on PubMed. Regarding the latter, we applied a combination of the search terms “cancer/NGS or WES (whole exome sequencing) or WGS (whole genome sequencing)” to confirm the number of clinical trials relating to cancer genomic medicine on ClinicalTrials.gov.

### 2.3. Comparative Analysis

Based on the information collected from the above research and analyses, this study sought to confirm whether MSK-IMPACT had made a certain contribution to promoting innovation in clinical sequencing in oncology. To ensure the fairness of the research, a comparative analysis between MSK-IMPACT, FoundationOne CDx, and Oncomine Dx Target Test was performed to gain objective insights.

First, this study investigated the characteristics of MSK-IMPACT and other tests to better understand if they have particular foundations to promote scientific research for innovation, which would help pharmaceutical companies conduct clinical trials and develop new cancer therapies.

**Figure 1:**
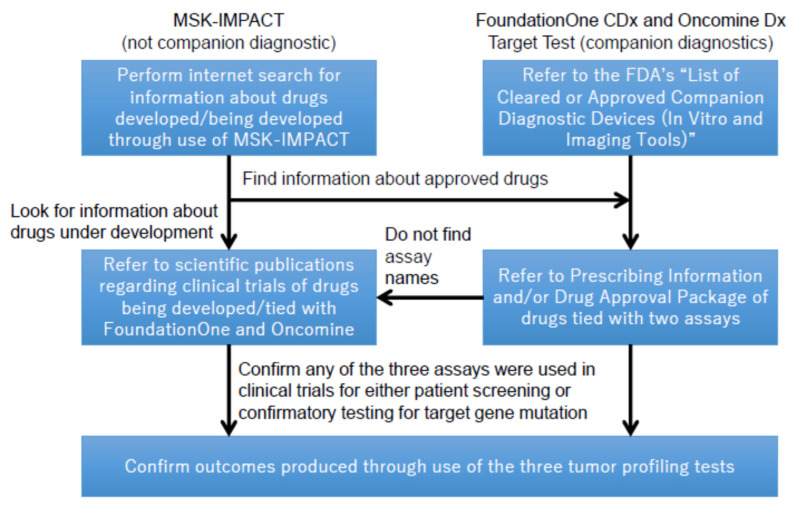
Investigation methods to define contribution to new drug development by the three tumor profiling tests.

Second, it also investigated whether these tests helped in the facilitation of healthcare innovation, particularly analyzing whether new drugs were successfully developed based on the use of such tests. For better clarification, this study defined the outcomes of the tests as ”drugs identified using three panel tests as a result of either patient screening or confirmatory testing of gene expressions upon the onset of clinical trials.” Overall, it appeared to be difficult to fully cover such outcomes in this study. As of 30 November 2020, ClinicalTrials.gov suggested only 2 observational studies through a keyword search using a single search term “MSK-IMPACT” [18]. Since observational studies were not considered as clinical trials, the search result did not indicate that the test had led to the development of new drugs for cancer treatment. Following this result, and due in part to the difficulties in accessing certain information on the outcomes of the tests, this study took different approaches to investigate the outcomes of MSK-IMPACT and those of the other tests, as illustrated in Figure 1. It then examined whether the new drugs among these outcomes identified by this investigation method utilized any of the FDA’s expedited approval programs using a drug development database Cortellis.com (https://www.cortellis.com/intelligence/home.do, accessed on 24 April 2021). This was intended to confirm the impact of the FDA’s regulatory reforms on the outcomes of each test.

### 2.4. Interview-Based Analysis

A semi-structured interview was conducted with an anonymous expert, the president of a company that provided its customers with clinical sequencing services, such as analytical services using NGS and tumor profiling tests, including MSK-IMPACT. The interview was focused on 3 key topics: (1) FDA’s regulatory reforms that have promoted the utilization and early application of new technologies in a clinical setting, (2) accumulation of publicly accessible open data on genetic information and its contribution to the development of new therapies in oncology, and (3) benefits and challenges of MSK-IMPACT in comparison with those associated with other tumor profiling tests from an innovation point of view. The interview was conducted for 1 h on 8 May 2020.

## 3. Results

### 3.1. FDA’s Regulatory Reforms and Their Outcomes

**Figure 2:**
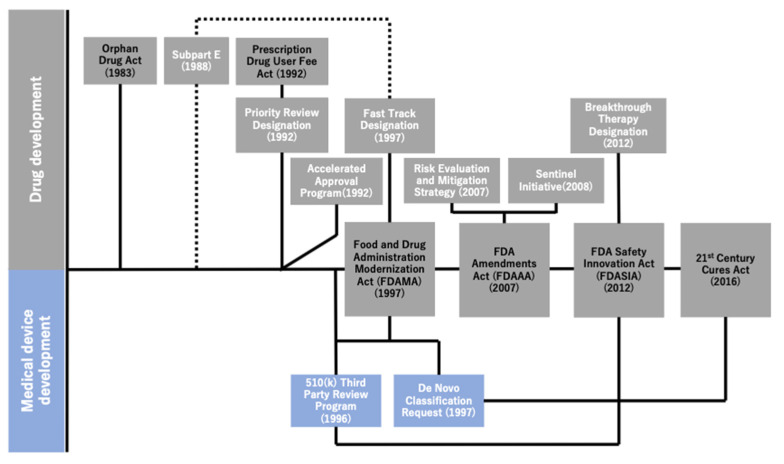
Historical overview of regulatory reforms by the U.S. Food and Drug Administration (FDA). This figure illustrates the association between the four major acts and relevant regulatory programs, actions, etc. in chronological order. The four major acts are lined up on the center line and are tied up with essential regulatory programs and actions implemented under any of such acts (i.e., the FDASIA and the Breakthrough Therapy Designation). The association between the subpart E regulations and the Fast Track Designation is expressed using the dotted lines because the former is the predecessor of the latter.

Figure 2 illustrates the historical overview of the regulatory reforms implemented by the FDA over the last three decades. As explained earlier, the series of regulatory reforms implemented by the FDA began after the enactment of the Orphan Drug Act of 1983, followed by that of expedited programs as well as other relevant acts to promote comprehensive healthcare innovation. The FDA Modernization Act (FDAMA), which was enacted in 1997 to reduce the review time for new drug candidates by extending the PDUFA of 1992, also sought to cover the medical devices. Meanwhile, the FDA intended to balance the risks between the early approval of new drugs and lack of scientific data. Under the FDA Amendments Act of 2007, the Risk Evaluation and Mitigation Strategy (2007) and Sentinel Initiative (2008) were implemented [10]. These programs were implemented to promote the safe use of medications [10,19] and mitigate risks by monitoring data regarding the adverse effects of drugs in certain patient populations [20]. Equally important was that the 21st Century Cures Act [21] of 2016 had sought to promote the utilization of medical data for new drug development, which triggered the facilitation of data utilization and accumulation of data on genetic mutations obtained through clinical sequencing in oncology.

Not only did the FDA work on reforming the pharmaceutical regulations, but it also performed practical actions to modernize the regulations for medical devices. This study was only focused on the regulations that can be considered to have facilitated the approval process of MSK-IMPACT. By employing the combination of the De Novo pathway and 510(k) 3 PR Program, both of which were implemented under the FDAMA, the FDA approved MSK-IMPACT, taking lesser time than originally envisioned. The FDA saved time by approving the test as an IVD in 51 days, which was shorter than the 150 days duration [22] originally set by the organization as the performance goal for the review of De Novo applications (through an email query to the FDA on 24 November 2020, we have additionally confirmed that “150 days” was the performance goal for the FDA De Novo reviews). It was also remarkable to note that these regulations allowed the test, which was originally considered as a Laboratory Developed Test (LDT), to be approved as an IVD. Prior to the implementation of these programs, there was no formal IVD approval process for LDTs; these were merely not-for-sale products developed in laboratories certified by the Clinical Laboratory Improvement Amendments and were not allowed to be distributed for commercial purposes.

Furthermore, to realize precision medicine, the FDA held public workshops twice in 2015 to take practical measures to establish regulations for clinical testing based on the utilization of NGS, gathering various stakeholders, including the College of American Pathologists, National Institutes of Health (NIH), National Institute of Standards and Technology, and Centers for Disease Control (CDC), as well as those from academia and manufacturers of diagnostic tools and instruments [23,24]. Based on a series of discussions, the FDA released the guidance draft to establish a regulatory pathway for cancer genomic medicine in 2016 [25]. Further, referring to the public comments, the FDA released certain guidelines in 2018, which summarized issues of how the FDA would interpret the clinical validity and significance of a product upon consideration of its regulatory approval [26].

### 3.2. The Contribution of Open Databases

Datasets of genetic information have been accumulated over time and were disclosed to the public in parallel due to the large-scale collaborative research programs triggered by the political will, combined with the advancement in DNA sequencing and analytical technologies. In 1999, the National Center for Biotechnology Information (NCBI) collaborated with the National Human Genome Research Institute to establish dbSNP, a data-sharing platform that provides genetic data on single nucleotide polymorphisms (SNPs) [27]. The International HapMap Project, which began in 2003, allowed researchers to analyze the reference dataset using the Genome-Wide Association Study (GWAS), a method that enabled the analysis of the association between diseases and relevant SNPs along with quantitative traits. The utilization of the reference dataset, encouraged by the establishment of the analytical tool, has contributed to the radical increase in the number of scientific publications [28]. In fact, according to the search results yielded using the search term “genome wide association study” on PubMed, the number of scientific publications relating to GWAS increased from 1 to 1808 between 2002 and 2018 [29]. Similarly, as a result of a keyword search using the search term “SNP” on PubMed, the number of SNP-related publications was also shown an increase from 721 in 2002 to 3826 in 2018 [29]. Such an accumulation in scientific knowledge of the association between diseases and SNPs eventually fueled the practical application of relevant technologies to a clinical setting; typical examples included tumor profiling tests and direct-to-consumer genetic testing services [28].

The barrage of scientific outcomes was reinforced by the practical application of NGS technologies after the launch of the world’s first NGS instrument in 2005. Some international collaborative research programs started using NGS, and the data obtained from these research programs were publicly released; the Genome Reference Consortium, the Personal Genome Project, and the 1000 Genome Project are some of the examples of such programs [30,31]. Data on genetic information obtained and accumulated from, both, basic research and clinical applications were further utilized. The NCBI has developed a data sharing platform by integrating different datasets with each other; it has become a foundation for further scientific research on and clinical applications of genetic testing [32]. Researchers are obliged to register data obtained from research programs supported by the NIH. Nevertheless, the platform has become popular among the global scientific community due to its user-friendliness. In the meantime, the rising tide of data disclosure further spilled over into the field of oncology. The Cancer Genome Atlas, which started in 2007, released a dataset of 4,938,362 genetic mutations from 7042 cases in 2013, accounting for 30 types of cancers [33].

As these genetic-information-based datasets continuously accumulated, activities to secure and improve the analytical validity of such data were also conducted through these large-scale, multicenter research programs by standardizing NGS instruments, tools, analytical protocols, and overall infrastructure required for scientific research. The CDC also organized the Next-Generation Sequencing: Standardization of Clinical Testing (Nex-StoCT), and published recommendations for the utilization of NGS in a clinical laboratory setting in 2012, specifically focusing on (1) validation, (2) quality control, (3) proficiency testing, and (4) reference materials [34].

Considering all these facts, it is worth paying attention to the recent trends in the field of cancer genomic medicine; a search result obtained using multiple search terms on ClinicalTrials.gov showed that the number of clinical trials in this field has gradually increased from 1 in 2008 to 30 in 2018 [18].

### 3.3. An Early Application of the New Technologies

The application of cancer genetic testing to both research and clinical settings was accelerated by the FDA’s approvals for Oncomine Dx Target Test, MSK-IMPACT, and FoundationOne CDx as IVDs in 2017 [35,36,37]. The FDA then simplified the review process for additional biomarkers, which would be brought after the approval of these tests, by allowing the test developers to report claims “without an FDA submission [38].” The decision was made based on the FDA’s approach that genetic mutations would fall into one of the three different evidence levels in accordance with the clinical significance, and that these evidence levels would be continuously updated as the science advances [38]. Companion diagnostics (CDx) were categorized as “Level 1” [38]. This level requires a genetic mutation to provide the highest clinical significance to be considered as a biomarker on the basis of clinical trials incorporating either “patient outcomes” or “clinical concordance to a previously approved CDx”, along with “analytical validity” of the test for that mutation [38]. “Level 2” requires “analytical validity” and “clinical validity” of the test, which is typically “publicly available clinical evidence” [38]. “Level 3” merely requires “analytical validation” in combination with the minimal level of clinical significance, such as “peer-reviewed publications” and “in-vitro preclinical models [38].” Genetic mutations that are neither Level 1 or 2 are considered Level 3, and these are not considered as biomarkers [38].

Based on the concept of three-tiered clinical significance, the FDA has allowed the test developers to move a genetic mutation from Level 3 to 2 without an additional FDA submission, if it can be recognized within the clinical community based on the accumulation of clinical evidence [38]. In addition, not only has the FDA allowed for a genetic mutation that accounts for a specific cancer type to be considered as a biomarker, but it has also paved the way for its approval as a biomarker for other cancers that can result from the same mutation.

Aside from the FDA’s regulatory efforts to simplify the review process for biomarkers, MSK-IMPACT was used as an LDT at MSKCC even before it was granted the FDA approval as an IVD in 2017 as stated earlier. It should also be emphasized again that MSK-IMPACT was approved in an accelerated manner as a result of the FDA’s regulatory efforts to establish the regulatory pathways for LDTs as mentioned earlier.

### 3.4. The Utilization Structure of MSK-IMPACT

**Figure 3:**
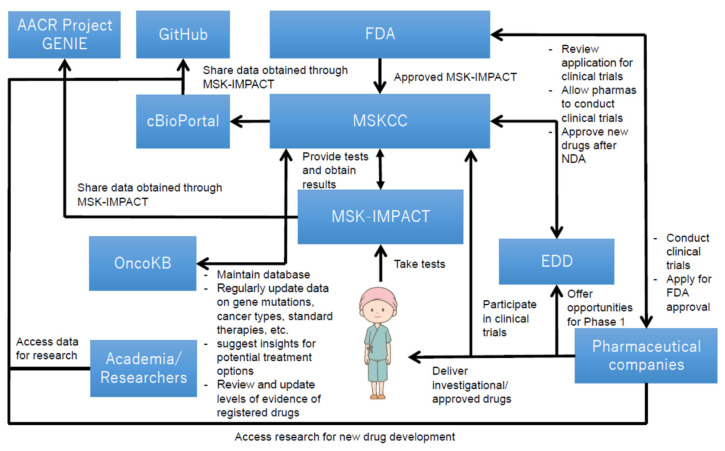
Key technological assets and their relationship in MSK-IMPACT [36,45,46]. Overall, this figure shows that the utilization structure of MSK-IMPACT contributes to both research and clinical settings. The structure helps genetic data obtained from cancer patients to be accumulated over time and be released to the public for further research. It also helps cancer patients participate in clinical trials. The structure has been established and reinforced based on interactions among various types of stakeholders (i.e., the FDA, MSKCC, cancer patients, pharmaceutical companies, etc.), and has provided research and clinical contributions, both of which are imperative for new drug development in oncology.

Figure 3 illustrates the overall structure of how MSK-IMPACT was utilized at MSKCC, which offers cancer care, diagnostic services, and opportunities for cancer patients to participate in the clinical trials in New York and New Jersey [39]. The utilization structure was gradually established as it was being used as an LDT. Genetic mutations data with clinical implications, collected from cancer patients, were accumulated and anonymously released to the public on “cBioPortal for Cancer Genomics” [40]. The hospital also developed an open source software that visualized data obtained through MSK-IMPACT; such data were released on GitHub to the public, and researchers are allowed to access them for free to facilitate further research for the development of novel therapeutic options in combination with the data released on cBioPortal [41,42]. Further, the spillover effect stemmed from the utilization of open data generated by MSK-IMPACT was found in a case of The Hyve B.V. (Utrecht, The Netherlands), a company that has developed free public software for cBioPortal [43]. Their software allows researchers to use data released on the data sharing platform [43]. Moreover, MSKCC has also established “OncoKB,” a knowledge base that helps healthcare professionals determine therapies based on the diagnostic outcomes provided by MSK-IMPACT [44]; this knowledge base, in accordance with the evidence levels regularly updated by the FDA, constantly updates information and data that are beneficial for decision-making for cancer therapies, such as those on cancer genetic mutations, cancer types, and molecular target drugs that can be potentially used for cancer treatment.

MSKCC has also established multiple processes to facilitate clinical trials by efficiently recruiting eligible patients in a timely manner, utilizing data collected through the applications of MSK-IMPACT. The hospital promotes phase 1 clinical trials by encouraging the treating physicians to introduce the Early Drug Development (EDD) Service to the eligible patients [45], which were identified by the DARWIN Cohort Management System, an original informatics platform used for the screening and management of patient cohorts for “genotype-matched clinical trials” [47]. In fact, the test suggested approximately 30% of patients would be eligible for clinical trials among more than 10,000 cancer patients in a study in which the clinical utility of MSK-IMPACT was evaluated using sequencing data obtained from such patients [48]. Furthermore, the hospital has recently initiated the Program for Drug Development in Leukemia (PDD-L) to promote the development of leukemia treatments by inducing leukemia patients to enroll in phase 1 clinical trials [49].

MSKCC has also functioned to conduct “basket trials,” which cover various cancer types by focusing on a specific genetic mutation that is considered to cause tumors [50]. Rather than focusing on a specific cancer type, basket trials enable researchers and drug developers to simultaneously cover the patients with different types of cancers [50]. In such a setting, rare cancers, for which the patient populations were generally small, can also be covered [50]. Vemurafenib (ZELBORAF^®^) was developed through a basket trial. The drug was first approved in August 2011 for the treatment of unresectable or metastatic melanoma associated with the *BRAF* V600 mutation [51]. MSKCC further provided an additional opportunity to conduct a basket trial to test the drug for *BRAF* V600 mutation-positive nonmelanoma patients [52]. As a result, the FDA approved the drug for the treatment of Erdheim–Chester disease (ECD), an extremely rare cancer, in November 2017 [53,54].

### 3.5. Comparison between MSK-IMPACT and Other Panel Tests

The results of the comparative analyses are shown in Table 2, Table 3 and Table 4. Table 2 summarizes the basic information regarding MSK-IMPACT, FoundationOne CDx and Oncomine Dx Target Test. The remarkable difference between these three assays is that MSK-IMPACT was not approved as a companion diagnostic assay, while its competing IVDs were listed as FDA-approved companion diagnostic devices [55]. The other difference was found in their data management systems; FoundationOne CDx and MSK-IMPACT appeared to have their own data sharing platforms, while OncomineCDx Target Test was merely found to possess its data management system, which would not be intended for data sharing with others. Second, Table 3 reveals the contribution of these three assays to new drug development. FoundationOne CDx appeared to be the most prominent, while Oncomine Dx Target Test, the other companion diagnostic device, seemed to have struggled to produce certain outcomes. In addition, there were 24 FDA-approved drugs associated with FoundationOne CDx for cancer care, while the number of such drugs for Oncomine Dx Target Test remained at 5 [55]. Although there were no CDx-tied drugs with MSK-IMPACT, it helped in the production of two FDA-approved drugs and two other drug candidates, which are currently under development. The other finding was that three of these drugs were identified through basket trials. Third, Table 4 shows the expedited approval programs that were helpful in obtaining FDA approvals for the new drugs produced based on the use of each panel test; considering the significance as well as difficulties of innovation, this study only focused on the new drugs, and thus excluded the existing drugs with history of expanding additional indications. It should be noted that these new drugs were found to have utilized multiple programs to accelerate the drug development process. Moreover, the average time frame between IND and FDA approval for these drugs was found to be approximately 3.5 years, which was significantly shorter than approximately 8 years that averaged from 1986 to 2017 as explained earlier [10].

## 4. Discussion

### 4.1. Implications of Regulatory Reforms to Corporate Activities

The number of outcomes produced by each panel test implies that FoundationOne CDx has benefited from the FDA’s regulatory reforms, early application of new technologies, and accumulation of publicly accessible open data. It would be reasonable to assume that the FDA has encouraged drug developers to facilitate drug development activities through the implementation of a series of regulatory reforms, including expedited approval programs. In the meantime, pharmaceutical regulations have become stringent in monitoring the safety of drug candidates under the FDA Amendments Act. The FDA’s strategies to balance the flexibility and stringency in drug development should be considered to be a reasonable action because the efficacy and safety of new therapies need to be secured and appropriately balanced, especially when such therapies are developed based on the utilization of the new technologies. It would also be reasonable to assume that data accumulation and disclosure to the public, along with the efforts for standardization and compatibility development between different datasets, has facilitated drug development activities in which FoundationOne CDx was incorporated. On the contrary, MSK-IMPACT does not seem to have fully benefitted from these regulatory efforts, although it succeeded in shortening the FDA’s review process for its IVD approval.

This may be because of the differences in the organizational interests and incentives between the developers. Foundation Medicine falls under the umbrella of the pharmaceutical giant Roche Holding AG (Basel, Switzerland), while MSKCC is a hospital. There is no doubt that the former has an interest in expanding collaborations with other players, such as pharmaceutical companies, to facilitate drug development activities using its products, considering the relationship with its parent company. On the other hand, the primary interest of MSKCC, as a healthcare provider, is to serve its patients.

In addition, the number of outcomes by Oncomine Dx Target Test was found to be inadequate despite its CDx approval. At this point, the fact that Life Technologies is a manufacturer of laboratory tools and is not directly involved in the drug development activities may account for this result. Therefore, it is reasonable to assume that, unlike the relationship between Foundation Medicine and Roche, the capital relationship of Life Technologies with its parent company, Thermo Fisher Scientific Inc. (Waltham, MA, USA), which is not a pharmaceutical company, has not functioned enough to motivate the company to be involved in new drug development.

### 4.2. Characteristics of MSK-IMPACT

Our study has identified tangible and intangible values of MSK-IMPACT. First, the test has been embedded into the patient recruitment activities of MSKCC for efficient enrollment in clinical trials. Second, there is an established utilization structure of data for genetic mutations in cancer patients collected using the test, which can be used for further research. Third, although the extent of the contribution of the test to basic cancer research has yet to be clear, the case of The Hyve, a free software developer, implies that MSK-IMPACT is believed to have contributed to basic research through its data sharing platform cBioPortal in combination with The Hyve’s free software. It is likely that researchers have gained some benefits from these tools as they can access the open data for free. This case represents the differentiation of MSK-IMPACT from FoundationOne CDx, which provides similar benefits at the researchers’ expense, such as the provision of data on a closed basis. Fourth, MSKCC and MSK-IMPACT have functioned as a catalyst to promote the practice of basket trials, which are an advanced form of clinical trials. Lastly, the test has thus far contributed to the development of both monotherapies and combination therapies for cancer. In contrast to FoundationOne CDx, the advantages associated with MSK-IMPACT were mostly identified in its integrated utilization structure within the MSKCC community.

Key challenges of MSK-IMPACT were pointed out from a marketing and business development point of view, considering the potential differences between this test and FoundationOne CDx. First, the test was basically used within the MSKCC community. This seems to have caused limitations for the test in gaining utilization opportunities outside the hospital group. Since the hospital has a well-established utilization structure of the test within its own community with a specific priority of saving patients, it has struggled to expand opportunities for the test to be used at other hospitals. The hospital may have also missed alliance and collaboration opportunities with other counterparts, such as pharmaceutical companies, for drug development activities. Second, cancer patients at MSKCC do not have to pay test fees because they are covered by donations [88]. This casts a concern about the sustainability of the testing practice. Since MSK-IMPACT has limitations in expanding marketing opportunities outside the MSKCC community, the hospital may have to consider alternative measures to ensure the sustainability of the testing practice for its patients.

### 4.3. Recommendations for a Better Clinical Sequencing in Oncology

Based on these considerations, we emphasize the importance of collaborations with external organizations, including other hospitals and pharmaceutical companies, for a non-profit model such as MSKCC to better promote drug development. Mirnezami et al. 2012 have pointed out that collaboration between various healthcare stakeholders, such as the governments, researchers, and pharmaceutical industries, would be required to promote precision medicine [89]. Looking at the comparison between MSK-IMPACT and FoundationOne CDx, the number of outcomes produced by the latter seems to be overwhelming, due in part to its CDx approval. The potential interest in drug development activities between Roche and Foundation Medicine should have been the major driving force. The difference in organizational interests can affect one’s motivation to facilitate innovation and even its consequences. As a case of collaborative development of cancer drugs, Makino et al. 2018 argued in their quantitative research that there was a positive correlation between the number of alliances (i.e., R&D licensing, marketing licensing, etc.) and a number of patents relating to CDx [90]. This implies that a challenge for MSK-IMPACT is to promote collaborative opportunities with external counterparts for drug development activities.

Despite these issues, MSKCC has established the utilization structure of MSK-IMPACT over time. Patients with cancer at MSKCC can easily be notified regarding their eligibility for clinical trials. Data on genetic mutations in patients at the hospital can also be utilized for further research. These processes can both, directly and indirectly, contribute to saving patients. Based on these findings, this study insists that even more patients would be saved if the characteristics of these two tests were to be mixed. It is recommended that MSKCC considers reinforcing collaborations with other hospitals, pharmaceutical companies, and the like and providing relevant resources to them to promote drug development activities.

Equally importantly, regulatory authorities need to consider establishing a certain institutional framework that integrates different healthcare stakeholders to facilitate drug development activities. For example, in the field of cell and gene therapy in Japan, a double-track regulation of providing values through medical services based on translational research and products based on clinical trials has been implemented, guaranteeing a variety of opportunities for companies and non-profit institutions [91]. Such an innovative approach in regulatory science will provide more opportunities for cancer drug development, which will eventually contribute to providing more treatment options for cancer patients.

### 4.4. Study Limitations

This study had some potential limitations. First, ClinicalTrials.gov did not function to accurately find clinical trials that employed MSK-IMPACT for either screening or confirmatory purposes, as pointed out earlier. Second, the investigation method to find outcomes by FoundationOne CDx and Oncomine Dx Target Test was not intended to cover ongoing clinical trials for their pre-approval drugs, while it detected some for MSK-IMPACT. Third, the method was not intended to cover the outcomes of basket trials by FoundationOne CDx and Oncomine Dx Target Test, while it found that the majority of the outcomes of MSK-IMPACT were developed through this form of clinical trials. Since the study focused on investigating the CDx-tied drugs with these two tests, the results did not convey the extent to which they were being used in the basket trials. Overall, the fact that numerous clinical trials involving cancer clinical sequencing have already been conducted accounts for the difficulties in fully covering the outcomes of these three tests. At this point, there is still room for further research to investigate the contribution of these three tests to cancer care innovation.

## 5. Conclusions

The present study explored factors that contribute to facilitating innovation in cancer clinical sequencing with a particular focus on the case of MSK-IMPACT with two comparative cases, FoundationOne CDx and Oncomine Dx Target Test. Through comparative analyses between these three tests, FoundationOne CDx appeared to have outweighed the MSK-IMPACT and Oncomine Dx Target Test in terms of the number of generated outcomes, whereas MSK-IMPACT was functioning as a hub to efficiently enroll cancer patients in clinical trials with its in-house data management platform. These results suggest two key challenges that MSK-IMPACT needs to overcome. First, more collaborations with external organizations for drug development activities, including but not limited to other hospitals and pharmaceutical companies, need to be pursued. Another challenge lies in the sustainability of the testing practice: since the use of the test is limited within the MSKCC community, it is ideal for the hospital to secure alternative financial sources to ensure continued testing practice. To address these challenges, MSK-IMPACT should expand the use of the test for collaborations with external organizations to develop novel cancer therapies. It should also be noted from a regulatory perspective that pharmaceutical regulations need to be supportive of drug developers, while balancing the efficacy and safety of new therapies under development in an appropriate manner. All these efforts will eventually contribute to the development of novel therapies for cancer patients.

## Figures and Tables

**Table 1:** Major stakeholders and catalysts.

Category	Stakeholders/Catalysts	Major Roles in Cancer Care Innovation
Regulatory authority	U.S. Food and Drug Administration (FDA)	Take responsibilities to set out and periodically reform pharmaceutical and medical devices regulations
Assay developer	Foundation Medicine, Inc., Life Technologies Corporation, Memorial Sloan Kettering Cancer Center (MSKCC)	Develop and commercialize tumor profiling tests
Developer of public data sharing platform	National Center for Biotechnology Information (NCBI), National Human Genome Research Institute	Establish and provide access to publicly accessible open data through international research programs/projects
International research program/project	Cancer Genome Atlas, Genome Reference Consortium, International HapMap Project, Personal Genome Project, 1000 Genome Project	Help researchers obtain genetic information from cancer patients
Drug manufacturer	Pharmaceutical companies (i.e., Roche Holding AG, Basel, Switzerland)	Develop new drugs and/or add new indications to the existing drugs on the basis of the use of tumor profiling tests
Healthcare institution	Hospitals providing healthcare services (i.e., MSKCC)	Provide cancer patients with opportunities for cancer care and clinical trials
Direct beneficiary of healthcare innovation	Cancer patients	Provide genetic data and use newly developed cancer therapies through clinical trials

**Table 2:** Comparison of basic information.

Item	FoundationOne CDx [37,55,56,57]	MSK-IMPACT [36,45,46,58]	Oncomine Dx Target Test [35,55,59,60]
Developer	Foundation Medicine, Inc.	Memorial Sloan Kettering Cancer Center (MSKCC)	Life Technologies Corporation
Date of FDA approval as IVD	30 November 2017	15 November 2017	22 June 2017
Specimen type	FFPE tumor tissue	FFPE tumor tissue and patient-matched blood/normal tissue as a normal control	FFPE tumor tissue
Number of genes covered	324	468	23
Biomarker	SNVs, Indels, CNVs, gene rearrangements, TMB, MSI and HRD	SNVs, Indels, CNVs, Promoter mutation (*TERT*), Gene rearrangements, TMB and MSI	SNVs, Deletions and Fusion gene (*ROS1*)
FDA approval for CDx	Granted for diagnosis of breast cancer, cholangiocarcinoma, colon/rectum cancer, non-small cell lung cancer (NSCLC), malignant melanoma, ovary cancer, prostate cancer, and solid cancer	None	Granted for diagnosis of non-small cell lung cancer (NSCLC)
Availability and functions of data management and/or sharing platform for new drug development	Allows access to open data on cancer patients through FoundationInsights, a cloud-based data platform. Provides access to FoundationCore through FoundationInsights, a knowledgebase with data obtained from cancer patients	Facilitates research for the development of new therapies through cBioPortal for Cancer Genomics (open database) Provides and updates clinical data obtained from cancer patients through OncoKB (knowledgebase) Allows patients to access Phase 1 clinical trials for solid tumors identified by the DARWIN Cohort Management System Manages study cohorts for clinical trials on a timely basis	Analyzes and reports sequencing data through the Torrent Suit Dx Software, which works on Google Chrome browser Allows sequencing results and reports to be automatically archived to an external server

The abbreviations for the terminology in genome science indicated in this table originally stand for the following: formalin fixed paraffin embedded (FFPE), single nucleotide variant (SNV), insertion/detection (Indel), copy number variation (CNV), tumor mutational burden (TMB), microsatelite instability (MSI), homologous recombination deficiency (HRD).

**Table 3:** Comparison of outcomes.

Product Name	New Drugs				Expanded Additional Indications to Existing Drugs			
	Drug Name	Biomarker	Indication/Therapy Type	Status	Drug Name	Biomarker	Indication/Therapy Type	Status
FoundationOne CDx	PEMAZYRE^®^ (pemigatinib) [61,62]	*FGFR2*	Cholangiocarcinoma/Monotherapy	Approved (April 2020)	GILOTRIF^®^ (afatinib) [63,64]	*EGFR*	Squamous cell carcinoma (lung)/Monotherapy	Approved (April 2016)
	ROZLYTREK^®^ (entrectinib) [65,66]	*NTRK*	Solid tumors/Monotherapy	Approved (August 2019)	KEYTRUDA^®^ (pembrolizumab) [67,68]	TMB	TMB-H solid tumors/Monotherapy	Approved (June 2020)
		*ROS1*	Non-small cell lung cancer (NSCLC)/Monotherapy		LYNPARZA^®^ (olaparib)	*BRCA1/2*	Ovarian cancer/Monotherapy [69,70]	Approved (December 2018)
	TABRECTA™ (capmatinib) [71,72]	Mutation relating to MET exon 14 skipping	Non-small cell lung cancer (NSCLC)/Monotherapy	Approved (May 2020)		HRR genes	mCRPC/Monotherapy [73,74]	Approved (May 2020)
	VITRAKVI^®^ (larotrectinib) [75,76]	*NTRK*	Solid tumors/Monotherapy	Approved (November 2018)	ZELBORAF^®^ (vemulafenib) [52,54]	*BRAF* V600	Erdheim-Chester disease (ECD)/Monotherapy	Approved (November 2017)
MSK-IMPACT	AZD5363 (capivasertib) [18,77]	*AKT1/2/3*	Multiple indications (breast cancer, prostate cancer, solid tumors, etc.)/Either monotherapy or combination	Phase 1~ (as of April 2021)	NERLYNX^®^ (neratinib) with XELODA^®^ (capecitabine) [77,78]	*HER2*	Breast cancer/Combination	Approved (February 2020)
	LOXO-195 (selitrectinib) [18,79]	*NTRK*	Solid tumors (with resistance to Larotrectinib)/Monotherapy	Phase 1/2 (as of April 2021)	-	*-*	-	-
	VITRAKVI^®^ (larotrectinib) [75,76]	*NTRK*	Solid tumors/Monotherapy	Approved (November 2018)	-	*-*	-	-
Oncomine Dx Target Test	GAVRETO™ (pralsetinib) [80,81]	*RET*	Non-small cell lung cancer (NSCLC)/Monotherapy	Approved (September 2020)	TAFINLAR^®^ (dabrafenib) with MEKINIST^®^ (trametinib) [82]	*BRAF* V600 E	Non-small cell lung cancer (NSCLC)/Combination	Approved (June 2017)

**Table 4:** Association between the FDA’s expedited approval programs and the new drugs identified by the three tumor profiling tests. To a greater or lesser extent, all the new drugs identified by the tests were found to have succeeded in speeding up the review process by utilizing a combination of the expedited approval programs.

Drug Information					Expedited Approval Programs [83]				
Drug Name (Generic Name)	Assay Used for Clinical Trials	Indication	IND Submission Date	Approval Date	Orphan Drug	Fast Track	Breakthrough Therapy	Priority Review	Accelerated Approval
GAVRETO™ (pralsetinib)	Oncomine Dx Target Test	Non-small cell lung cancer (NSCLC)	August 2019 [84]	September 2020 [81]	Yes	No	Yes	Yes	Yes
PEMAZYRE^®^ (pemigatinib)	FoundationOne CDx	Cholangiocarcinoma	January 2018 [85]	April. 2020 [62]	Yes	No	Yes	Yes	Yes
ROZLYTREK^®^ (entrectinib)	FoundationOne CDx	Solid tumors	February 2014 [83]	August 2019 [66]	Yes	No	Yes	Yes	Yes
		Non-small cell lung cancer (NSCLC)	May 2017 [65]	August 2019 [66]	Yes	No	No	Yes	Yes
TABRECTA™ (capmatinib)	FoundationOne CDx	Non-small cell lung cancer (NSCLC)	January 2015 [86]	May 2020 [72]	Yes	No	Yes	No	Yes
VITRAKVI^®^ (larotrectinib)	FoundationOne CDx, MSK-IMPACT™	Solid tumors	February 2014 [87]	November 2018 [76]	Yes	No	Yes	Yes	Yes

## Data Availability

Our study did not generate any numerical data.
